# Myo-ODE: continuous-time trajectory reconstruction and risk prediction of high myopia via neural ordinary differential equations

**DOI:** 10.3389/fpubh.2026.1897882

**Published:** 2026-07-09

**Authors:** Na Zhao, Runze Zheng, Jinhao Lu, Zhaoyu Huang, Cairui Li, Chao Dai, Xiao Enbei, Jian Wang

**Affiliations:** 1 School of Software, Yunnan University, Kunming, China; 2 Department of Ophthalmology, People's Hospital of Dali Bai Autonomous Prefecture, Yunnan, China; 3 College of International Students & Preparatory Education, Sichuan University, Chengdu, China; 4 College of Information Engineering and Automation, Kunming University of Science and Technology, Kunming, China

**Keywords:** childhood myopia, continuous-time modeling, dynamic simulation, myopia progression, neural ordinary differential equations, risk stratification

## Abstract

The global surge in childhood myopia necessitates robust screening tools for early risk stratification; however, conventional predictive models often struggle with irregular follow-up intervals and fail to capture the continuous nature of refractive development. We propose Myo-ODE, a continuous-time framework based on Neural Ordinary Differential Equations (Neural ODEs) to predict high myopia risk. Unlike traditional discrete machine learning models, Myo-ODE parameterizes the derivative of the refractive state, allowing myopia progression to be represented as a continuous latent dynamic flow. This architecture explicitly accommodates non-uniform screening intervals and supports temporal interpolation and cautious short-term extrapolation within the observed follow-up horizon. Evaluated on a longitudinal dataset (*N* = 4,973), Myo-ODE achieved the highest F1-score of 0.8000 (95% CI: 0.7741–0.8256) and Recall of 0.7812 (95% CI: 0.7518–0.8103), while maintaining a competitive AUC of 0.9834 (95% CI: 0.9781–0.9887). Furthermore, our framework reconstructs individualized refractive progression trajectories and provides an interpretable estimate of the model-learned progression momentum of SE change. By bridging the gap between discrete clinical observations and continuous trajectory-level modeling, Myo-ODE offers a promising tool for personalized myopia surveillance in real-world screening environments.

## 1 Introduction

Myopia has emerged as a major global public health challenge, with projections suggesting that 50% of the world's population will be affected by 2050 (1). This burden is particularly pronounced in East Asia, where rapid educational, environmental, and behavioral changes have coincided with high rates of childhood-onset myopia. The clinical concern is not limited to blurred distance vision: high myopia (SE ≤ −6.0D) substantially increases the lifetime risk of irreversible visual impairment due to myopic macular degeneration, retinal detachment, glaucoma, and other structural complications (2, 3). Because refractive status in childhood can change rapidly over a short period, school-based vision screening provides an important opportunity to identify children whose refractive trajectories may require closer surveillance or timely referral.

Despite the increasing availability of longitudinal screening data, translating repeated measurements into individualized risk estimates remains difficult. Conventional screening workflows usually classify students according to their current spherical equivalent (SE) or visual acuity at a single visit, which may overlook children who have not yet crossed a high-risk threshold but are progressing quickly. Recent machine learning and deep learning studies have shown promise for predicting myopia onset, progression, or high-myopia risk from demographic, ophthalmic, behavioral, and fundus-imaging variables (4–11). However, many such models are optimized for fixed follow-up windows and point-wise classification rather than for reconstructing each child's underlying refractive trajectory, although recent time-aware and AI-based models have begun to address longitudinal visit timing more explicitly (12–14). Prior longitudinal and AI-based myopia studies have increasingly recognized that visit timing and follow-up duration influence prediction performance. For example, recent models using routine eye-examination records, multifactorial risk profiles, or dynamic biometric information have begun to incorporate longitudinal measurements, variable observation windows, or time-aware predictors when estimating myopia onset and progression risk (10, 11, 13, 14). These studies provide important evidence that temporal information improves individualized risk assessment. However, most existing approaches still represent time through discrete prediction horizons, engineered interval features, or fixed-window summaries, rather than learning a continuous transition function that can be evaluated over arbitrary follow-up intervals.

A fundamental limitation in current myopia predictive modeling is the temporal discretization bias. Traditional machine learning (ML) paradigms—ranging from Logistic Regression to advanced ensemble methods such as XGBoost—typically treat myopia progression as a sequence of static, independent snapshots (15, 16). However, refractive development is inherently a continuous biological process governed by complex ocular growth dynamics. By forcing continuous trajectories into discrete time bins, conventional models can lose information about the pace and direction of change, including the trajectory-level progression signal that may distinguish slow progressors from children who are rapidly approaching high myopia.

Furthermore, real-world school screenings are frequently affected by the irregular sampling problem. Unlike controlled clinical trials, longitudinal school data often feature non-uniform follow-up intervals due to student absenteeism, transfer between schools, equipment availability, or logistical shifts in screening schedules. Discrete-time models struggle to handle these temporal gaps without resorting to crude interpolation, mean-interval approximation, or data exclusion, each of which can introduce bias and reduce clinical utility in resource-limited or unpredictable screening environments. Recent evidence that lifestyle disruptions, outdoor exposure, parental myopia, baseline refractive state, behavioral routines, regional epidemiology, and school-screening features can all contribute to risk prediction further suggests that robust models should flexibly integrate heterogeneous predictors across visits rather than assume a single complete feature vector at a fixed time point (4, 5, 12, 17–20). A clinically useful model should therefore represent both the current refractive state and the elapsed time between observations.

To bridge these gaps, we propose Myo-ODE, a continuous-time framework for myopia progression based on Neural Ordinary Differential Equations (Neural ODEs) (21). Our approach shifts the modeling paradigm from discrete point-estimation to latent dynamic flow simulationl. By parameterizing the derivative of the refractive state with a neural network, Myo-ODE learns the continuous mathematical representation of refractive-state change. This enables the framework to reconstruct smooth, individualized trajectories from sparse, irregularly sampled observations and to perform temporal interpolation or cautious extrapolation within clinically plausible follow-up horizons. In this way, Myo-ODE is designed not only to classify high-myopia risk, but also to provide an interpretable estimate of model-estimated progression momentum that can support longitudinal surveillance.

The primary contributions of this study are as follows:

- **Continuous-time trajectory reconstruction:** to our knowledge, we introduce one of the first Neural ODE frameworks for myopia progression modeling, conceptualizing refractive development as a continuous dynamical system rather than a set of discrete transitions.
- **Resilience to irregular follow-ups:** we demonstrate that by learning the infinitesimal dynamics of refractive change, Myo-ODE explicitly handles non-uniform screening intervals, maintaining predictive stability where fixed-interval ML models experience performance degradation.
- **Interpretability via learned continuous dynamics:** we provide a model-based view of refractive progression by visualizing learned continuous latent dynamics. This provides a mathematical indicator of progression momentum, offering a dynamic complement to static spherical equivalent (SE) values.
- **Validated performance on real-world cohorts:** validated on a longitudinal dataset of 4,973 students from Binchuan County, China, Myo-ODE achieved the highest F1-score and Recall among the compared models, with a competitive AUC of 0.9834.

## 2 Materials and methods

### 2.1 Framework overview

**Figure 1:**
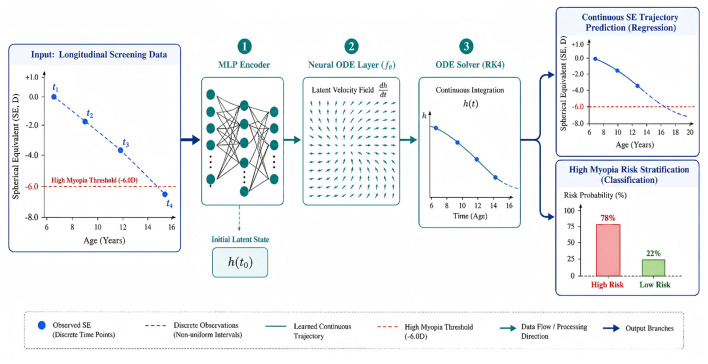
Overall architecture of the proposed Myo-ODE framework. The pipeline converts irregular longitudinal screening histories into continuous latent trajectories and then produces simultaneous spherical-equivalent regression and high-myopia risk classification outputs.

The overall pipeline of the proposed Myo-ODE framework is illustrated in Figure 1. The architecture consists of three core components designed to process longitudinal data with non-uniform sampling intervals, model continuous-time refractive trajectories, and deliver robust multi-task clinical predictions.

As shown in the left panel of Figure 1, the input layer ingests sparse, irregularly sampled screening histories summarized by demographic variables, baseline spherical equivalent (SE), elapsed follow-up time, and pre-outcome trajectory features such as Δ*SE* and annualized SE progression rate. Unlike conventional sequential models that enforce rigid, uniform time steps, Myo-ODE directly accommodates arbitrary time gaps (*t*_1_, *t*_2_, …, *t*_*n*_) without temporal alignment or aggressive imputation.

The core architecture, shown in the middle panel, formalizes refractive development as a continuous latent dynamic flow. First, an MLP encoder compresses the leakage-free pre-outcome observations into a low-dimensional latent state *h*(*t*_0_), representing the student's baseline and historical refractive profile. Subsequently, a specialized neural network, *f*_θ_, parameterizes the derivative of the latent state with respect to time ($\frac{dh}{dt}$), which can be conceptually interpreted as a learned continuous mathematical representation of refractive progression momentum rather than a directly observed biological growth rate. To reconstruct the continuous-time trajectory, an Ordinary Differential Equation (ODE) solver—specifically utilizing the fourth-order Runge-Kutta (RK4) method—integrates this continuous vector field over the observed and queried temporal horizons.

Finally, as depicted in the right panel, the integrated continuous latent trajectory is passed to dual output heads to execute simultaneous tasks. The regression branch reconstructs a smooth, individualized SE trajectory over observed or queried time points, while the classification branch evaluates the probability of the student crossing the clinically critical high-myopia threshold ( ≤ −6.0D), outputting risk stratification for referral consideration.

### 2.2 Study population, measurements, and prediction task

This longitudinal school-based study used repeated vision-screening data collected in Binchuan County, Dali Prefecture, Yunnan Province, China, from Autumn 2023 to Autumn 2025. The source data consisted of preexisting retrospective records from a routine school-based public-health vision-screening program jointly conducted by Dali Prefecture People's Hospital and primary and secondary schools in Binchuan County. The screening was part of school health service and public-health surveillance rather than an interventional research procedure. Batch-level school-screening records were first cleaned at the record level and then linked into student-specific longitudinal trajectories using anonymized unique student identifiers. The formal modeling unit was the individual student rather than the screening record. This student-level unit was chosen because the intended use case is population-level school screening and referral prioritization rather than eye-level clinical diagnosis.

Record-level cleaning removed duplicate entries, conflicting records, records missing key identity-linkage information, and records with incomplete core ocular measurements. After cleaning, records from different screening batches were linked by anonymized student ID to reconstruct each student's longitudinal refractive history.

Students were eligible for inclusion if they were 6–15 years of age, with the final cohort ranging from 6.16 to 15.99 years, thereby covering primary and junior-secondary school ages. Eligible students were required to have at least three consecutive historical screening records before the outcome-defining follow-up record, so that longitudinal trajectory features could be constructed without using future outcome information. Students also had to have complete key ophthalmic data, including uncorrected distance visual acuity (UCDVA), refractive measurements, and computable spherical equivalent (SE).

Students with pre-existing ocular pathologies, including amblyopia, strabismus, or organic eye diseases, or a history of refractive surgery were excluded to ensure that the modeled trajectories reflected natural refractive development rather than secondary ocular conditions or surgical correction. Physiologically implausible measurements were removed or manually reviewed as potential testing artifacts, including *SE*>+10.0D, *SE* < −15.0D, and five-point visual acuity chart scores below 3.0. Extreme annualized refractive shifts were also reviewed as potential systematic measurement errors.

After record-level cleaning, student-level linkage, age eligibility screening, consecutive-follow-up requirements, data-integrity assessment, ocular pathology or surgery exclusion, and physiological plausibility filtering, the final individual-level modeling cohort consisted of 4,973 unique students. Records were de-identified before analysis, and student identifiers were replaced by anonymous study codes prior to model development. Ethical approval and consent procedures are summarized in the Ethics Statement.

#### 2.2.1 Clinical measurements and data pre-processing

Ocular examinations were performed by trained ophthalmic technicians following a standardized school-screening protocol. Non-cycloplegic refraction was conducted using an automated infrared refractor under consistent measurement conditions. For each eye, spherical power, cylindrical power, and axis were recorded. The Spherical Equivalent (SE) was calculated using the standard formula: *SE* = Sphere+0.5 × Cylinder. Unless otherwise specified, analyses used the average SE across both eyes for each screening visit to provide a single student-level refractive state. High myopia was defined as average *SE* ≤ −6.00 D at the final follow-up, while milder severity categories were defined according to conventional SE thresholds. Because refraction was non-cycloplegic, potential accommodative bias was considered when interpreting absolute SE values, especially among younger students (22, 23). We used average binocular SE to obtain a stable student-level refractive summary aligned with public-health screening workflows and to reduce the influence of isolated eye-level measurement noise. The student-level average binocular SE was explicitly designated as the screening-risk outcome metric, rather than an eye-level diagnostic endpoint, because administrative triage, guardian notification, and referral follow-up within large-scale school screening programs are executed at the student level. We acknowledge that this aggregation may dilute unilateral or asymmetric high myopia; nevertheless, it aligns the algorithm's predictions with the actionable logistical unit of public-health intervention. Future implementations should evaluate dual-eye or eye-specific input channels for clinically targeted referral decisions.

Batch-level descriptive records were not interpreted as the final number of unique students. The available screening records by round were 2,434 in Autumn 2023, 4,555 in Spring 2024, 4,716 in Autumn 2024, 4,318 in Spring 2025, and 393 in Autumn 2025 (Table 1). Descriptive batch-level longitudinal profiles of ocular development and myopia escalation are summarized in Table 2. Because the same student could contribute records in multiple screening batches, these batch-level counts are descriptive record counts and cannot be directly summed or compared with the final individual-level modeling cohort of 4,973 unique students.

**Table 1:** Available batch-level screening records by screening round.

Screening round	Available screening records
2023 Autumn	2,434
2024 Spring	4,555
2024 Autumn	4,716
2025 Spring	4,318
2025 Autumn	393

These counts represent usable screening records within each batch and are distinct from the final individual-level modeling cohort (*N* = 4, 973 unique students).

**Table 2:** Descriptive batch-level longitudinal profiles of ocular development and myopia escalation across representative screening rounds.

Clinical metrics	Baseline (2023)	Mid-term (2024)	Final (2025)	Net change	Annual rate	*P*-value^*^
1. Refractive components (D)						
Mean spherical equivalent (SE)	−0.77 ± 1.47	−1.30 ± 1.93	−1.37 ± 2.10	−0.60	−0.468 ± 1.73	< 0.001
Right eye sphere (S)	−0.43 ± 1.49	−1.08 ± 1.90	−1.19 ± 2.04	−0.76	–	< 0.001
Left eye sphere (S)	−0.33 ± 1.45	−0.90 ± 1.85	−0.98 ± 1.95	−0.65	–	< 0.001
Cylinder (astigmatism)	−0.78 ± 0.71	−0.62 ± 0.69	−0.57 ± 0.75	+0.21	–	< 0.05
2. Visual function (UCDVA)						
Right eye (five-point chart score)	4.81 ± 0.32	4.70 ± 0.39	4.67 ± 0.41	−0.14	–	< 0.001
Left eye (five-point chart score)	4.81 ± 0.32	4.72 ± 0.38	4.69 ± 0.40	−0.12	–	< 0.001
Visual impairment rate (< 5.0)	42.3%	57.1%	59.2%	+16.9%	–	< 0.001
3. Myopia progression (%)						
Overall prevalence	42.28%	59.80%	52.32%^†^	+10.04%	–	< 0.001
Mild myopia prevalence	34.02%	43.60%	32.99%	–	–	–
Moderate myopia prevalence	7.15%	13.44%	15.26%	+8.11%	–	–
High myopia prevalence	1.11%	2.76%	4.07%	+2.96%	–	–
4. Demographic slice						
Mean age (years)	9.68 ± 2.12	11.44 ± 2.56	12.43 ± 2.93	+2.75 y	–	–
Gender (male ratio)	48.4%	48.4%	48.4%	–	–	–

^*^Repeated-measures ANOVA for continuous variables; chi-square test for categorical variables.

^†^Prevalence drop in final batch is due to cohort composition shift (fewer high school samples). These batch-level summaries should be interpreted separately from the final individual-level modeling cohort (*N* = 4, 973 unique students).

Data pre-processing was performed before model training to ensure temporal consistency and reduce avoidable noise. Records with missing key identifiers, UCDVA, refractive information, or variables required to compute SE were excluded from the primary analysis. Continuous variables were standardized using statistics estimated from the training folds only, and the same transformations were then applied to validation and test folds to avoid information leakage. Categorical variables such as gender were encoded as model inputs using a fixed coding scheme.

To prepare the data for continuous-time modeling, all screening timestamps were converted into a relative time-span format *t*_*ij*_ (years), where *t*_*i*0_ = 0 represents the baseline visit for student *i* and *j* = 1, …, *m*_*i*_ indexes subsequent visits. The elapsed time between visits was computed from the actual screening dates rather than rounded to an annual interval. Unlike traditional models that require fixed intervals, our framework preserves the exact decimal follow-up duration for each student, allowing the ODE solver to account for irregular sampling and variable observation gaps.

#### 2.2.2 Prediction task definition and leakage prevention

The primary task was defined as binary classification of future high-myopia risk at a pre-specified outcome-defining follow-up record, rather than classification of current high-myopia status at the input visit. For student *i*, let *T*_*i*_ denote the time of the outcome-defining follow-up record and let $H_{i}=\left\{j:t_{ij}<T_{i}\right\}$ denote all historical screening records available before that outcome time. The input to the model was constructed exclusively from $H_{i}$, including baseline screening variables and longitudinal trajectory-derived features.

The outcome label was defined only from the outcome-defining follow-up record: *y*_*i*_ = 1 if the average SE at *T*_*i*_ was ≤ −6.00D and *y*_*i*_ = 0 otherwise. Here, *T*_*i*_ was treated as the pre-specified prediction horizon or queried follow-up time available to the model; only the time coordinate was used during prediction, whereas the SE measurement at *T*_*i*_ was reserved for label definition and evaluation. Trajectory features such as Δ*SE* and annualized SE progression rate were computed only from pre-outcome screening intervals; when two historical time points were used to estimate progression, both time points were required to occur before *T*_*i*_. Specifically, for each prediction instance, Δ*SE* was calculated as the difference between the earliest available historical SE and the most recent pre-outcome SE, and the annualized progression rate divided this change by the elapsed time between those two historical visits. For horizon-stratified analyses, the same rule was applied within the available pre-outcome window: the queried endpoint defined *T*_*i*_, and no ocular measurement from *T*_*i*_ or any later visit contributed to the trajectory features. For example, for a 12-month prediction horizon, the endpoint visit defined the prediction target and label, while only visits chronologically preceding that endpoint were used to construct baseline trajectory summaries such as Δ*SE* and annualized progression rate. Any follow-up records beyond the prognostic window were masked during feature engineering to eliminate post-outcome data leakage. The outcome-defining follow-up record itself was not used to calculate Δ*SE*, annualized progression rate, imputation values, normalization parameters, feature-selection decisions, model hyperparameters, or probability thresholds. Operationally, Myo-ODE receives baseline screening variables together with the pre-outcome longitudinal trajectory and outputs a future high-risk or low-risk stratification for the subsequent follow-up endpoint.

### 2.3 Myo-ODE architecture and optimization

The core of our methodology is the Myo-ODE framework, which represents myopia progression as a continuous trajectory in a model-based latent space. The architecture consists of four primary components: a feature encoder, a neural ODE function, an ODE solver, and task-specific regression and classification heads. The encoder summarizes baseline and pre-outcome trajectory information into an initial latent state; the ODE function learns how that state evolves over continuous time; the numerical solver propagates the latent state to each observed or queried follow-up time; and the prediction heads convert the latent state into refractive-error and high-myopia-risk estimates.

This design differs from a conventional recurrent or feed-forward model because the same learned transition function can be evaluated at arbitrary time intervals. Therefore, a student observed after 0.8 years and another observed after 1.3 years are not forced into the same discrete step. Instead, both trajectories are generated by integrating the learned dynamics over their actual elapsed times. This property is central to the intended use of Myo-ODE in school-screening settings where visit schedules are informative but irregular.

#### 2.3.1 Latent state encoding

Each student *i* is initially represented by a leakage-free pre-outcome feature vector (Equation 1):


$$\begin{array}{c} u_{i}=\left[Age_{i},Gender_{i},SE_{i,0},\Delta SE_{i},r_{i},\Delta t_{i}\right], \end{array}$$ (1)

where Δ*SE*_*i*_ denotes the SE change between historical screening visits, *r*_*i*_ = Δ*SE*_*i*_/Δ*t*_*i*_ denotes the annualized SE progression rate, and Δ*t*_*i*_ denotes the elapsed time between the historical visits used to compute this progression summary. These trajectory-derived quantities were computed only from records in $H_{i}$ before the outcome-defining time *T*_*i*_. An encoder network *E*_ϕ_, implemented as a multi-layer perceptron (MLP), maps these baseline and pre-outcome trajectory features into an initial latent state $h_{i}\left(0\right)\in {\mathbb{R}}^{d}$ (Equation 2):


$$\begin{array}{c} h_{i}\left(0\right)=E_{\phi }\left(u_{i}\right) \end{array}$$ (2)

This latent state ***h***_*i*_(0) serves as the “initial condition” for the subsequent differential equation, representing the student's baseline and historical refractive profile.

#### 2.3.2 Continuous-time dynamics via neural ODEs

We define the evolution of the latent state ***h***_*i*_(*t*) as an Ordinary Differential Equation (ODE). Instead of manually specifying the functional form, we parameterize the derivative using a neural network *f*_θ_ (Equation 3):


$$\begin{array}{c} \frac{dh_{i}\left(t\right)}{dt}=f_{\theta }\left(h_{i}\left(t\right),t\right) \end{array}$$ (3)

where *f*_θ_ represents the learned continuous latent dynamics of refractive-state change. This network consists of three fully connected layers with Softplus activation functions to ensure that the learned vector field is continuous and differentiable, while avoiding assumptions about the underlying causal mechanism of eye growth. Accordingly, this quantity should be interpreted as a model-derived mathematical representation of progression momentum, not as a directly measured axial elongation rate or biological growth velocity.

#### 2.3.3 Numerical integration and prediction

To obtain the predicted state at any future time *T*, we solve the initial value problem (IVP) using a numerical ODE solver (Equation 4):


$$\begin{array}{c} h_{i}\left(T\right)=h_{i}\left(0\right)+\int_{0}^{T}f_{\theta }\left(h_{i}\left(t\right),t\right)dt \end{array}$$ (4)

In this study, we employed the Fourth-order Runge-Kutta (RK4) method for deterministic integration over the observed follow-up times. To support efficient training on the full cohort (*N* = 4, 973) while maintaining constant memory usage with respect to solver depth, gradients were computed using the Adjoint Sensitivity Method during backpropagation, as commonly adopted in Neural ODE implementations. The final latent state ***h***_*i*_(*t*_*ij*_) is mapped to the predicted refractive error ${\hat{SE}}_{i}\left(t_{ij}\right)$ via a linear regressor *D*_ψ_ and to the high-myopia probability *p*_*i*_(*t*_*ij*_) via a sigmoid classification head *g*_ω_ (Equation 5):


$$\begin{array}{c} {\hat{SE}}_{i}\left(t_{ij}\right)=D_{\psi }\left(h_{i}\left(t_{ij}\right)\right),\;p_{i}\left(t_{ij}\right)=\sigma \left(g_{\omega }\left(h_{i}\left(t_{ij}\right)\right)\right). \end{array}$$ (5)

#### 2.3.4 Optimization and loss functions

The Myo-ODE framework is optimized end-to-end to reconstruct historical SE trajectories while preserving final high-myopia risk discrimination. The primary classification target was future high-myopia risk, defined by the final follow-up label *y*_*i*_ = ⊮(*SE*_*i*_(*T*_*i*_) ≤ −6.0D). To avoid outcome leakage, all trajectory-derived predictors, including annualized SE progression rate and Δ*SE*, were computed only from screening intervals preceding the outcome assessment time point. The final outcome visit was used to define *y*_*i*_ and to evaluate prediction, but not to construct pre-outcome input features or validation-fold thresholds. For the auxiliary SE reconstruction objective, only historical observations available before the prediction target time were used in the supervised regression term during risk-model training. To avoid scale imbalance between the refractive-error term and the unitless classification term, the SE residual is normalized by the training-fold standard deviation *s*_*SE*_. The loss is Equation 6:


$$\begin{array}{r} L=\lambda_{SE}\frac{1}{\underset{i}{\sum }|H_{i}|}\overset{N}{\underset{i=1}{\sum }}\underset{j\in H_{i}}{\sum }{\left(\frac{{\hat{SE}}_{i}\left(t_{ij}\right)-SE_{i}\left(t_{ij}\right)}{s_{SE}}\right)}^{2}\\ +\lambda_{cls}\frac{1}{N}\overset{N}{\underset{i=1}{\sum }}\text{CE}\left(y_{i},p_{i}\left(T_{i}\right)\right), \end{array}$$ (6)

where $H_{i}=\left\{j:t_{ij}<T_{i}\right\}$ denotes pre-outcome historical visits and CE denotes binary cross-entropy. The relative task weights λ_*SE*_ and λ_*cls*_ are selected within the cross-validation workflow using validation-fold performance, with F1-score prioritized for the high-myopia classification task.

### 2.4 Robustness, statistical analysis, and implementation

To evaluate sensitivity to irregular follow-up timing, we performed a Temporal Stress Test. We simulated real-world follow-up inconsistencies by introducing random delays δ∈[0, 0.5] years to the follow-up timestamps in the test folds. Discrete models used fixed-interval or mean-interval approximations, whereas Myo-ODE used its internal solver to integrate across the perturbed interval *t*_*ij*_+δ. Because the perturbation does not create newly observed refraction labels at *t*_*ij*_+δ, this experiment should be interpreted as a time-domain stability analysis rather than a prospective validation at unobserved delayed visits.

#### 2.4.1 Sparse-visit, calibration, and temporal generalization analyses

To further assess robustness and implementation readiness, we conducted four additional validation analyses. First, a sparsity stress test was performed by randomly dropping 25%, 50%, and 75% of longitudinal follow-up observations from the test trajectories while preserving each student's baseline record. This experiment mimicked missed school screenings, absenteeism, and incomplete follow-up in real-world public health surveillance. The same trained models were then evaluated on the degraded test trajectories without retraining.

Second, probability calibration was evaluated using the Brier Score and Expected Calibration Error (ECE). For *M* probability bins *B*_*m*_, ECE was defined as Equation 7:


$$\begin{array}{c} \text{ECE}=\overset{M}{\underset{m=1}{\sum }}\frac{\left|B_{m}\right|}{n}|\text{acc}\left(B_{m}\right)-\text{conf}\left(B_{m}\right)|, \end{array}$$ (7)

where acc(*B*_*m*_) denotes the observed high-myopia frequency and conf(*B*_*m*_) denotes the mean predicted probability within bin *m*. Decision Curve Analysis (DCA) was used to quantify clinical utility across risk thresholds *p*_*t*_ by comparing the standardized net benefit of model-guided referral against “Treat All” and “Treat None” strategies (Equation 8):


$$\begin{array}{c} \text{Net Benefit}=\frac{TP}{n}-\frac{FP}{n}\times \frac{p_{t}}{1-p_{t}}. \end{array}$$ (8)

Third, to approximate prospective use, we performed temporal hold-out validation. Records collected from Autumn 2023 to Autumn 2024 were used for model training, and subsequent 2025 follow-up screening data were held out as an independent future cohort. This time-aware analysis was treated as a temporal robustness check and did not replace the primary student-level train–test evaluation. Finally, subgroup and horizon-stratified analyses were conducted by age group, sex, baseline refractive category, and prediction horizon to evaluate algorithmic stability across clinically relevant strata.

#### 2.4.2 Statistical analysis

Predictive performance was quantified using AUC-ROC, F1-score, precision, recall, and Mean Absolute Error (MAE). The primary analysis used an 80% training and 20% independent testing split at the student level, stratified by the final high-myopia outcome label. Each student's complete longitudinal trajectory was treated as an indivisible unit, so all historical and follow-up records from the same student were assigned exclusively to one subset.

Hyperparameter tuning and decision-threshold selection were performed only within the training set using five-fold cross-validation with student-level grouping. The same student could not appear in both the training and validation folds. Probability thresholds were selected from the training-set cross-validation procedure by maximizing validation F1-score and were fixed before application to the independent test set. The independent test set was held out until the final evaluation and was not used for preprocessing-parameter estimation, imputation, normalization, feature selection, model selection, hyperparameter tuning, or threshold selection. Core test-set metrics are reported as point estimates with 95% confidence intervals estimated by 1,000 nonparametric bootstrap resamples at the student level. Calibration was assessed with the Brier Score and ECE. Longitudinal changes in continuous SE were evaluated with repeated-measures or mixed-effects methods, whereas ordinal myopia-severity trends were assessed with methods appropriate for repeated observations. All experiments were conducted using PyTorch 2.1 and the torchdiffeq library.

#### 2.4.3 Implementation and reproducibility details

For reproducibility, all preprocessing operations, feature normalization parameters, model fitting, threshold selection, and evaluation were performed within the training data only and then applied unchanged to the held-out test set. The Myo-ODE encoder used a latent dimension of *d* = 16, and the ODE function used three fully connected layers with hidden width 64 and Softplus activations. The RK4 solver integrated over each student's observed relative screening times without rounding intervals to fixed annual steps.

Models were optimized with AdamW using an initial learning rate of 1 × 10^−3^, weight decay of 1 × 10^−4^, mini-batch size of 256 students, and a maximum of 300 epochs. Early stopping was applied if validation F1-score did not improve for 30 consecutive epochs. Class imbalance was handled by weighting the positive class in the binary cross-entropy term according to the high-myopia prevalence within the training fold. Probability thresholds were selected on the validation fold by maximizing F1-score and were then fixed before test-fold evaluation.

Baseline models were trained using the same student-level train–test split, the same grouped cross-validation procedure within the training set, and the same pre-outcome feature set. Hyperparameters for XGBoost, LightGBM, Random Forest, and MLP baselines were selected by validation-fold performance using predefined search grids. Test-set confidence intervals were estimated by 1,000 student-level bootstrap resamples within the held-out test set or temporal hold-out cohort. ECE was calculated using 10 equal-frequency probability bins, and DCA was evaluated over risk thresholds from 0.10 to 0.50.

## 3 Results

### 3.1 Overall performance and latent dynamics

We first evaluated the predictive accuracy of Myo-ODE in identifying students at risk of progressing to high myopia (SE ≤ −6.0D). Given the severe class imbalance (approx. 5% high myopia prevalence), we prioritized the F1-Score and Recall as primary metrics over Accuracy.

As summarized in Table 3, XGBoost achieved the highest AUC (0.9891; 95% CI: 0.9847–0.9932), whereas Myo-ODE achieved the highest F1-Score (0.8000; 95% CI: 0.7741–0.8256), Precision (0.8196; 95% CI: 0.7927–0.8461), and Recall (0.7812; 95% CI: 0.7518–0.8103). The F1-score improvement over XGBoost was 1.48 percentage points (approximately 1.88% relative improvement). Because the bootstrap confidence intervals of Myo-ODE and XGBoost overlapped, we interpret this gain as modest rather than definitive evidence of statistical superiority. Its potential clinical value lies mainly in the direction of improvement—higher recall and F1-score for a rare high-risk outcome—but this magnitude should be confirmed in larger external cohorts before being considered clinically decisive. This pattern suggests that the continuous integration process may be particularly useful for identifying high-risk fast progressors in an imbalanced screening setting, even when another model has a slightly higher AUC.

**Table 3:** Performance comparison across diverse machine learning architectures.

Model	AUC (95% CI)	F1-score (95% CI)	Precision (95% CI)	Recall (95% CI)
Random forest	0.9632 (0.9561–0.9704)	0.7334 (0.7048–0.7621)	0.7688 (0.7392–0.7983)	0.7012 (0.6685–0.7338)
XGBoost	**0.9891 (0.9847–0.9932)**	0.7852 (0.7584–0.8119)	0.8188 (0.7910–0.8462)	0.7544 (0.7245–0.7841)
LightGBM	0.9855 (0.9802–0.9903)	0.7721 (0.7449–0.7990)	0.8061 (0.7774–0.8343)	0.7410 (0.7108–0.7706)
Vanilla MLP	0.9774 (0.9708–0.9836)	0.7288 (0.6995–0.7580)	0.7654 (0.7352–0.7950)	0.6955 (0.6624–0.7283)
**Myo-ODE (ours)**	0.9834 (0.9781–0.9887)	**0.8000 (0.7741–0.8256)**	**0.8196 (0.7927–0.8461)**	**0.7812 (0.7518–0.8103)**

Values are independent student-level test-set point estimates with 95% confidence intervals from student-level bootstrap resampling. Bold values indicate the top-performing model configuration for each respective clinical evaluation metric.

#### 3.1.1 Neural latent dynamics and vector field analysis

**Figure 2:**
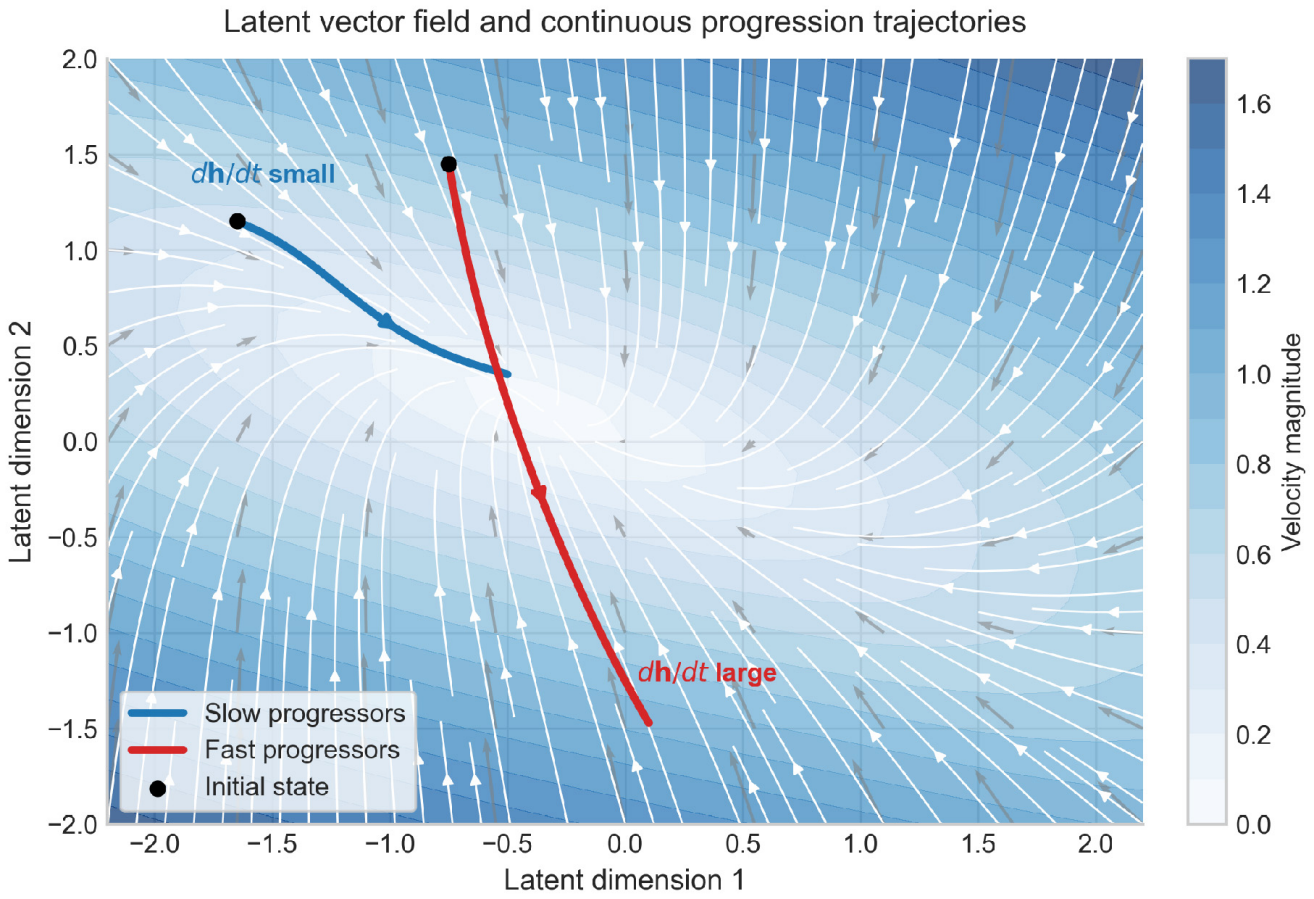
Learned latent dynamics and representative fast- vs. slow-progressor trajectories. The arrows summarize the model-estimated direction and magnitude of latent refractive-state change, while the overlaid trajectories illustrate distinct progression patterns learned by Myo-ODE.

To move beyond purely black-box prediction, we analyzed the learned continuous latent dynamics *f*_θ_(***h***_*i*_(*t*), *t*). This analysis provides a model-based view of how refractive change is represented as a latent dynamical system (Figure 2). These visualizations should be understood as model explanations in latent space, not as directly observed ocular-biometric measurements.

The learned derivative $\frac{dh_{i}\left(t\right)}{dt}$ was sensitive to the initial SE state and baseline age. For students in the “Fast Progressor” group, the model learned a steeper negative refractive-change trajectory, consistent with faster SE deterioration during the pre-pubertal myopic shift. Quantitative analysis revealed that for individuals aged 10–12 with an initial SE of −2.0D, the predicted annualized progression rate was −0.65 ± 0.15D/year. This ability to summarize model-estimated refractive progression momentum provides a dynamic complement to static SE values; however, because axial length was not measured, these trajectories should not be interpreted as direct measurements of axial elongation or biological growth velocity.

### 3.2 Robustness, calibration, and clinical utility

A core limitation of traditional ML models is their reliance on fixed-step transitions. We conducted a stress test to evaluate model resilience under simulated irregular follow-up intervals, where the second screening was delayed by 0.1 to 0.5 years.

The results showed that Myo-ODE maintained a Mean Absolute Error (MAE) of 0.18 ± 0.04D across the simulated delay scenarios. In contrast, XGBoost's MAE increased from 0.22D to 0.42D under longer simulated delays as the delay exceeded 0.3 years. Because this experiment used controlled timestamp perturbations rather than prospectively observed delayed-visit labels, we interpret the between-model difference as a descriptive degradation trend rather than as a stand-alone inferential test of clinical performance. These findings support the time-domain stability of the ODE formulation under perturbed follow-up intervals, but they should not be interpreted as proof of accuracy at unobserved future visits without prospective delayed-visit labels.

#### 3.2.1 Calibration and clinical utility

In addition to discrimination, we evaluated whether predicted probabilities were numerically reliable for risk communication and referral decisions. Myo-ODE achieved the lowest calibration error, with an ECE of 0.0124 and a Brier Score of 0.0842, whereas XGBoost and MLP showed higher ECE values of 0.0583 and 0.0612, respectively (Table 4). These results suggest that Myo-ODE's probability estimates were closer to the observed high-myopia event frequencies across risk strata.

**Table 4:** Probability calibration metrics for high-myopia risk prediction.

Model	ECE	Brier score
XGBoost	0.0583	0.1037
Vanilla MLP	0.0612	0.1124
**Myo-ODE (ours)**	**0.0124**	**0.0842**

Lower ECE and brier score indicate better calibration. Bold values indicate the top-performing model configuration for each respective clinical evaluation metric.

**Figure 3:**
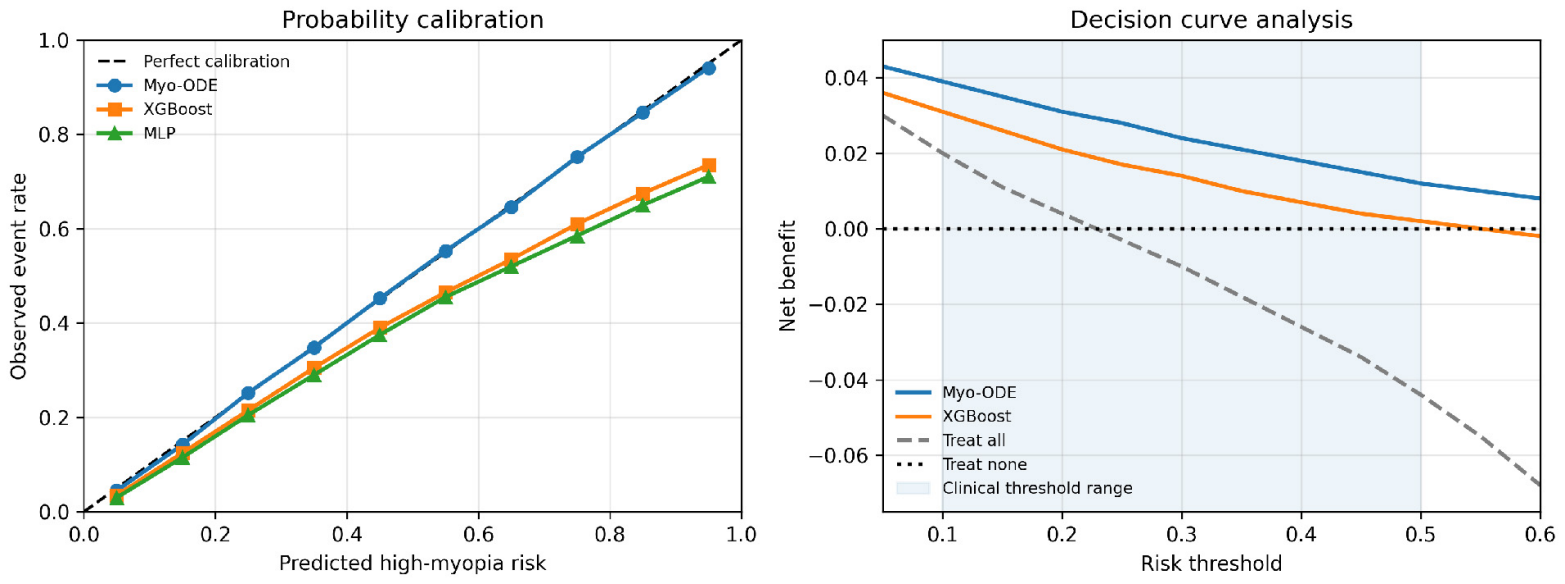
Calibration and clinical decision analysis. The panel **(left)** compares predicted high-myopia probability with observed event frequency, and the panel **(right)** shows Decision Curve Analysis across clinically relevant risk thresholds.

The calibration curve of Myo-ODE closely followed the 45-degree ideal calibration line, whereas the discrete baselines showed overconfidence in the high-risk region (>0.7). Decision Curve Analysis further showed that Myo-ODE showed generally higher net benefit than Treat All, Treat None, and XGBoost across much of the clinically plausible threshold range, with the most relevant advantage observed in the central referral-threshold region (Figure 3). In practical terms, a higher net benefit in this threshold range indicates that, for a given referral threshold, Myo-ODE may identify more students who will progress to high myopia while avoiding some unnecessary referrals compared with non-model-based strategies or the XGBoost comparator. Because decision curves can converge or cross at peripheral thresholds, these DCA results should be interpreted as evidence of potential clinical utility within the main screening decision window rather than as proof of uniform superiority at every possible threshold. These findings support the potential clinical utility of calibrated continuous-time risk estimates, while prospective decision-impact evaluation remains necessary before routine clinical use.

#### 3.2.2 Robustness to sparse follow-up records

We next evaluated whether Myo-ODE remained stable when longitudinal screening histories were incomplete. Under random removal of 25% and 50% of follow-up observations, Myo-ODE's F1-score decreased only from 0.8000 to 0.7842 and 0.7615, respectively. In contrast, XGBoost decreased from 0.7852 to 0.5920 at the 50% dropout level, indicating greater sensitivity to sparse follow-up histories. These sparse-visit results should be interpreted as a controlled perturbation analysis under the specified random dropout configuration rather than as an exhaustive characterization of all missing-data mechanisms. The observed pattern nevertheless suggests slower performance degradation for Myo-ODE under progressively reduced longitudinal observation density.

**Figure 4:**
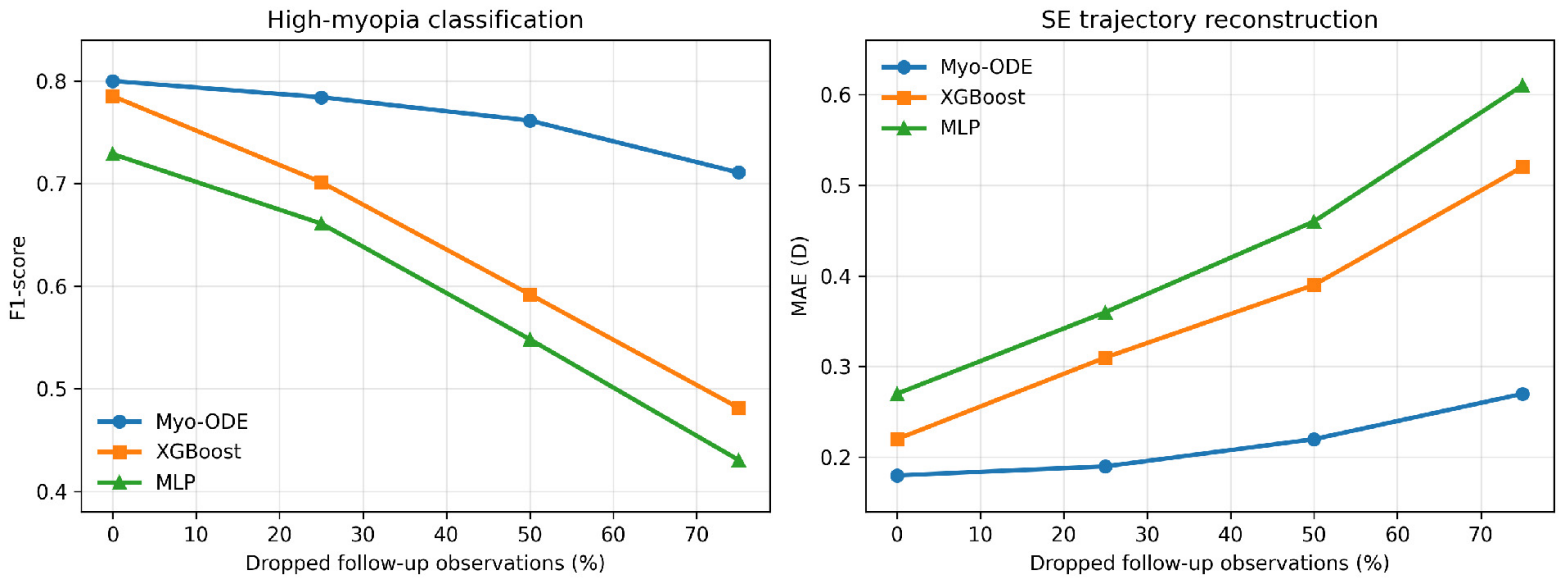
Sparse-visit robustness analysis under random follow-up dropout. Myo-ODE exhibits slower degradation in F1-score and MAE than XGBoost and MLP as longitudinal records become increasingly sparse.

At the extreme 75% dropout level, where many students retained only baseline and one follow-up observation, Myo-ODE maintained an F1-score of 0.7108. The corresponding MAE curve also increased more gradually than those of the discrete baselines (Figure 4). This stress test approximates missing completely at random follow-up loss and does not fully represent informative missingness due to absenteeism, transfer, or health-seeking behavior; nevertheless, it supports the robustness of the continuous-time formulation under sparse observation schedules.

### 3.3 Temporal, subgroup, and horizon-stratified validation

Before evaluating future-cohort performance, we compared the historical training cohort with the 2025 hold-out cohort to characterize temporal cohort shift. As shown in Table 5, the 2025 cohort had a slightly older age distribution and higher high-myopia prevalence than the historical training cohort. These differences support the use of temporal hold-out validation as a stricter assessment than random record-level partitioning.

**Table 5:** Comparison between the historical training cohort and the 2025 temporal hold-out cohort.

Characteristic	Historical training	2025 hold-out	*P*-value
Students, *n*	4,580	393	–
Age, years	11.18 ± 2.43	12.43 ± 2.93	< 0.001
Male, *n* (%)	2,217 (48.4)	190 (48.3)	0.97
Baseline SE, D	−1.24 ± 1.87	−1.37 ± 2.10	0.18
High-myopia cases, *n* (%)	156 (3.4)	16 (4.1)	0.46
Mean follow-up duration, years	1.42 ± 0.39	1.68 ± 0.31	< 0.001

Values summarize student-level records available before final outcome assessment.

Under this temporally separated evaluation, Myo-ODE achieved an AUC of 0.9712 (95% CI: 0.9580–0.9841), F1-score of 0.7854 (95% CI: 0.7428–0.8275), recall of 0.7625 (95% CI: 0.7084–0.8160), and MAE of 0.22D (95% CI: 0.18–0.26). The modest decline relative to the primary student-level test-set evaluation suggests temporal stability within this dataset, although this remains an internal temporal validation rather than an external validation in an independent region. Importantly, the 2025 hold-out cohort contained only 393 students and 16 high-myopia cases. Because bootstrap intervals estimated from such a small number of events can be sensitive to resampling variation, the F1-score, recall, and confidence intervals from this analysis should be viewed as exploratory evidence of temporal robustness rather than definitive prospective validation. In particular, recall estimates from this small-event cohort were interpreted together with the student-level bootstrap distribution rather than as a stable single-count sensitivity estimate from one discrete confusion matrix.

#### 3.3.1 Subgroup and horizon-stratified performance

We further examined whether performance was concentrated in specific demographic or refractive subgroups. Table 6 reports subgroup sample size, high-myopia positive cases, F1-score, and MAE. The positive-case counts in the temporal validation table and subgroup table should not be directly summed or compared because they refer to different analysis streams: the temporal validation cohort was defined by chronological training—hold-out eligibility, whereas subgroup analyses were based on the primary student-level evaluation framework with subgroup-specific completeness requirements. Therefore, differences in positive-case totals reflect analysis-specific cohort definitions rather than inconsistent outcome labeling. F1-scores remained within a narrow range across age, sex, and baseline SE strata, although the number of positive cases was limited in some subgroups and should be considered when interpreting apparent stability.

**Table 6:** Subgroup and prediction-horizon stratified performance of Myo-ODE.

Analysis	Stratum	*n*	Positive cases	F1-score (95% CI)	MAE (D)
Age	6–9 years	1,684	47	0.8062 (0.7620–0.8501)	0.17
Age	10–15 years	3,289	156	0.7895 (0.7602–0.8184)	0.19
Sex	Male	2,407	95	0.7928 (0.7581–0.8270)	0.18
Sex	Female	2,566	108	0.8074 (0.7740–0.8402)	0.18
Baseline SE	Emmetropia	1,128	28	0.7816 (0.7298–0.8332)	0.16
Baseline SE	Mild myopia	2,643	103	0.7989 (0.7650–0.8324)	0.18
Baseline SE	Moderate myopia	1,202	72	0.8103 (0.7692–0.8510)	0.20
Horizon	0–6 months	–	–	–	0.12 (0.10–0.14)
Horizon	6–12 months	–	–	–	0.16 (0.13–0.19)
Horizon	12–24 months	–	–	–	0.21 (0.17–0.25)

Subgroup rows report sample size and high-myopia positive cases to contextualize performance estimates.

The horizon-stratified results showed gradual error accumulation with longer prediction windows rather than abrupt degradation. This pattern is consistent with stable short-term trajectory reconstruction, but it should not be interpreted as unrestricted long-range extrapolation beyond the observed screening horizon.

### 3.4 Ablation, efficiency, and scalability

We performed an ablation study to quantify the impact of the ODE solver configuration on predictive stability. We compared the standard Myo-ODE (using a fourth-order Runge-Kutta solver) with a simplified Euler-based version and a discrete ResNet-like baseline.

As shown in Table 7, the transition from the discrete ResNet baseline to the full RK4 Myo-ODE increased F1-score by 5.88 percentage points (0.7412–0.8000). The Euler-based Myo-ODE also improved over the discrete baseline by 4.13 percentage points, while RK4 further refined MAE from 0.21D to 0.18D, suggesting that both the continuous-time parameterization and solver accuracy contributed to the final performance.

**Table 7:** Ablation analysis of ODE structural components.

Configuration	Solver	F1-score (95% CI)	MAE (D, 95% CI)
Discrete ResNet	N/A	0.7412 (0.7120–0.7701)	0.31 (0.28–0.35)
Myo-ODE (Simplified)	Euler	0.7825 (0.7554–0.8094)	0.21 (0.18–0.25)
**Myo-ODE (full)**	**RK4**	**0.8000 (0.7741–0.8256)**	**0.18 (0.15–0.22)**

Bold values indicate the top-performing model configuration for each respective clinical evaluation metric.

#### 3.4.1 Computational efficiency and scalability

Finally, we evaluated the feasibility of deploying Myo-ODE in large-scale screening systems. Through the use of mini-batch parallelization and optimized tensor operations, the training time for the entire cohort (*N* = 4, 973) was approximately 62 seconds in our local computational environment. The inference latency was measured at 1.85 ms per sample, allowing rapid generation of progression trajectories for large screening batches. Because runtime depends on hardware, threading, and solver settings, implementation reports should document the processor, memory configuration, batch size, and solver tolerances alongside these timing results.

## 4 Discussion

### 4.1 Summary of key findings

This study developed and validated Myo-ODE, a continuous-time trajectory reconstruction framework designed to predict high myopia risk in real-world screening environments. By parameterizing the derivative of refractive states using Neural Ordinary Differential Equations (Neural ODEs) (21), our model achieved the highest F1-score of 0.8000 and Recall of 0.7812 among the compared models, while XGBoost achieved the highest AUC. The main finding is therefore not that Myo-ODE dominates every metric, but that continuous-time modeling may improve clinically important high-risk detection under class imbalance and irregular follow-up timing within this cohort.

### 4.2 The power of continuity: why Myo-ODE helps under irregular follow-up

**Figure 5:**
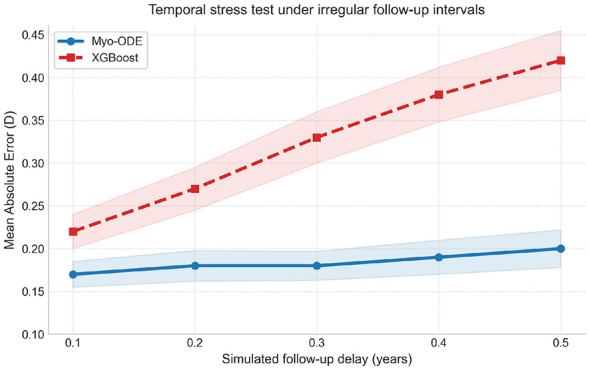
Temporal stress-test summary under simulated follow-up delays. Myo-ODE maintains a relatively stable MAE profile, whereas XGBoost shows increasing error under longer delays.

In our baseline implementation, discrete predictive models such as XGBoost and Random Forest encode follow-up information through fixed or summary features rather than through an explicit continuous-time transition model (24, 25). As shown in Table 3 and Figure 5, these models can perform strongly on fixed-interval datasets but are more sensitive to perturbations of the follow-up interval. This limitation is methodological rather than inherent to the tree algorithms themselves: without an explicit transition function over *dt*, the model has no built-in mechanism for integrating refractive change over arbitrary observation gaps.

In contrast, Myo-ODE operates by learning the continuous latent dynamics $\frac{dh_{i}\left(t\right)}{dt}=f_{\theta }\left(h_{i}\left(t\right),t\right)$. The learned vector field reflects model-estimated different rates of SE deterioration across baseline age and initial refractive state. For instance, the model identified a steeper negative refractive-change trajectory in the “Fast Progressor” group, consistent with the observed pre-pubertal myopic shift. By treating time as a continuous variable, Myo-ODE maintained a MAE of 0.18 ± 0.04D under simulated delayed screenings, whereas XGBoost's error increased under the same perturbation. This stress test supports time-domain robustness, although prospective validation with truly delayed follow-up labels remains necessary. Because the delay experiment used random perturbations, it may not fully capture informative missingness caused by absenteeism, school transfer, illness, differential health-seeking behavior, or local screening logistics; therefore, these findings should be interpreted as a controlled sensitivity analysis rather than definitive evidence of real-world missing-data robustness.

### 4.3 Robustness and scalability in public health surveillance

The ablation study (Table 7) supports the contribution of the ODE-based design. The full RK4 Myo-ODE improved F1-score by 5.88 percentage points relative to the discrete ResNet-like baseline, and the Euler version still improved F1-score by 4.13 percentage points. The RK4 solver further reduced MAE from 0.21D to 0.18D compared with the Euler version, suggesting that solver accuracy contributes to stable multi-year projections.

From an implementation-feasibility perspective, Myo-ODE showed low inference latency in the local testing environment. Nevertheless, runtime should be interpreted with hardware and implementation context, including CPU type, number of threads, solver tolerances, and batch size. This caveat is consistent with reporting recommendations for prediction models that use machine learning (26).

### 4.4 Comparison with existing literature

Most existing myopia prediction models focus on maximizing discrimination using complete or fixed-interval longitudinal records (9–11, 15, 16, 27, 28). Real-world screenings, however, are often affected by absenteeism and inconsistent follow-up windows. Common missing-data strategies such as multiple imputation by chained equations can reduce bias when data are missing under appropriate assumptions (29, 30), but they do not by themselves define a continuous-time refractive trajectory. Compared with prior time-aware myopia prediction approaches that incorporate follow-up duration as a covariate or summarize longitudinal change over predefined windows, Myo-ODE explicitly parameterizes the continuous-time evolution of the latent refractive state. This distinction allows the model to integrate over non-uniform observation gaps and to reconstruct individualized trajectories from sparse school-screening records, while still remaining compatible with conventional risk-classification endpoints. Thus, the proposed framework should be viewed as complementary to existing longitudinal prediction models, with a specific methodological emphasis on continuous-time trajectory reconstruction under irregular follow-up schedules.

Our work shifts the paradigm from point-wise prediction toward trajectory-based risk modeling. To our knowledge, Myo-ODE is among the first applications of Neural ODEs to myopia progression, using the mathematical structure of ODEs to model refractive change continuously over observed and perturbed follow-up times. This approach complements broader trends in clinical prediction modeling and AI reporting (21, 26). Nevertheless, because all data came from Binchuan County, model transportability to regions with different educational pressure, outdoor exposure, socioeconomic context, ethnicity, and screening practice remains unproven. The present study should therefore be regarded as a county-level methodological validation, and multicenter external validation across diverse socioeconomic regions is a necessary next step before broader public-health deployment.

### 4.5 Clinical implications and limitations

The ability of Myo-ODE to generate individualized model-estimated SE progression-momentum profiles is clinically relevant because it allows risk stratification to consider both current refractive status and rate of deterioration. Such a dynamic profile could help identify children who may benefit from closer monitoring even before they cross a high-myopia threshold. This is particularly important because recent work links refractive trajectories with ocular biometric changes and downstream pathological-myopia complications, highlighting the need for earlier trajectory-level surveillance rather than late-stage threshold detection (14, 31, 32).

Several limitations remain. First, refraction was measured without cycloplegia, so accommodative bias may affect absolute SE estimates, especially in younger children (22, 23). In large-scale school screening, universal cycloplegic refraction is difficult to implement because of time, staffing, safety monitoring, and parental-acceptance constraints. However, non-cycloplegic refraction may overestimate myopia in some younger children because of accommodation, potentially causing false-positive risk labels or boundary misclassification near the high-myopia threshold. Because this retrospective screening dataset did not include a cycloplegic refraction sub-cohort, we could not directly quantify the exact rate of high-myopia misclassification attributable to accommodative bias. Consequently, the observed SE trajectories and estimated risk dynamics should be interpreted as screening-level trends rather than definitive clinical diagnoses, necessitating formal cycloplegic confirmation in secondary clinical referrals. Therefore, the model should be interpreted as a school-screening risk-stratification tool rather than a substitute for cycloplegic refraction or comprehensive ophthalmic diagnosis. Second, the use of average binocular SE supports student-level public-health triage but may miss unilateral or asymmetric high myopia; eye-level outcomes and dual-channel bilateral modeling should be evaluated in future clinical implementations. Third, axial length was not available; therefore, the learned trajectories should be interpreted as refractive-change trajectories rather than direct evidence of axial elongation or causal biological mechanisms. This limitation reflects the practical constraints of routine school screening, where optical biometers are often unavailable or not cost-effective for full-population surveillance. Fourth, Myo-ODE does not currently incorporate environmental, spatial, or social factors such as classroom lighting, outdoor activity duration, parental myopia, or peer-group trends. The absence of these biological and environmental predictors limits mechanistic interpretation and may reduce generalizability across regions. Fifth, the temporal stress test is a perturbation analysis rather than a substitute for external prospective validation. Random dropout and random delay may overestimate robustness when missingness is informative. In addition, the 2025 hold-out set had only 16 high-myopia events, so temporal validation metrics should be interpreted cautiously. Future work should integrate multi-modal risk factors, report calibration metrics in detail, incorporate eye-level and axial-length outcomes where feasible, and validate the model in independent cohorts before clinical use.

### 4.6 Conclusion

The Myo-ODE framework represents a transition from fixed-interval prediction toward continuous-time modeling in school-based myopia surveillance. By bridging Neural ODEs with longitudinal refractive data, Myo-ODE provides a promising research framework for high-risk screening support under irregular follow-up schedules. The current evidence supports modestly improved F1-score and recall in this cohort, while external validation, calibration reporting, eye-level evaluation, cycloplegic confirmation pathways, and prospective delayed-visit evaluation are needed before broad clinical use.

## Data Availability

The raw data supporting the conclusions of this article will be made available by the authors, without undue reservation.
